# Differential Timing and Coordination of Neurogenesis and Astrogenesis in Developing Mouse Hippocampal Subregions

**DOI:** 10.3390/brainsci10120909

**Published:** 2020-11-26

**Authors:** Allison M. Bond, Daniel A. Berg, Stephanie Lee, Alan S. Garcia-Epelboim, Vijay S. Adusumilli, Guo-li Ming, Hongjun Song

**Affiliations:** 1 Department of Neuroscience and Mahoney Institute for Neurosciences, Perelman School of Medicine, University of Pennsylvania, Philadelphia, PA 19104, USA; ambond1@pennmedicine.upenn.edu (A.M.B.); daberg@pennmedicine.upenn.edu (D.A.B.); sylee@sas.upenn.edu (S.L.); garciaal@sas.upenn.edu (A.S.G.-E.); ViajaySubbaRa.Adusumili@pennmedicine.upenn.edu (V.S.A.); gming@pennmedicine.upenn.edu (G.-l.M.); 2 Department of Cell and Developmental Biology, Perelman School of Medicine, University of Pennsylvania, Philadelphia, PA 19104, USA; 3 Institute for Regenerative Medicine, University of Pennsylvania, Philadelphia, PA 19104, USA; 4 Department of Psychiatry, Perelman School of Medicine, University of Pennsylvania, Philadelphia, PA 19104, USA; 5 The Epigenetics Institute, Perelman School of Medicine, University of Pennsylvania, Philadelphia, PA 19104, USA

**Keywords:** neurogenesis, astrogenesis, hippocampus, cytogenesis, birth-dating

## Abstract

Neocortical development has been extensively studied and therefore is the basis of our understanding of mammalian brain development. One fundamental principle of neocortical development is that neurogenesis and gliogenesis are temporally segregated processes. However, it is unclear how neurogenesis and gliogenesis are coordinated in non-neocortical regions of the cerebral cortex, such as the hippocampus, also known as the archicortex. Here, we show that the timing of neurogenesis and astrogenesis in the Cornu Ammonis (CA) 1 and CA3 regions of mouse hippocampus mirrors that of the neocortex; neurogenesis occurs embryonically, followed by astrogenesis during early postnatal development. In contrast, we find that neurogenesis in the dentate gyrus begins embryonically but is a protracted process which peaks neonatally and continues at low levels postnatally. As a result, astrogenesis, which occurs during early postnatal development, overlaps with the process of neurogenesis in the dentate gyrus. During all stages, neurogenesis overwhelms astrogenesis in the dentate gyrus. In addition, we find that the timing of peak astrogenesis varies by hippocampal subregion. Together, our results show differential timing and coordination of neurogenesis and astrogenesis in developing mouse hippocampal subregions and suggest that neurogenesis and gliogenesis occur simultaneously during dentate gyrus development, challenging the conventional principle that neurogenesis and gliogenesis are temporally separated processes.

## 1. Introduction

Many fundamental principles of mammalian brain development were derived from studies of neocortical development. Early during neocortical brain development, neuroepithelial cells in the ventricular zone divide symmetrically to expand the precursor population [1]. Then, neuroepithelial cells transform into radial glia cells, the neural stem cells of the developing brain, which divide asymmetrically to generate differentiated progeny [2]. Radial glia cells first generate neurons and then undergo a fate switch to generate glia [3,4]. This neurogenic-to-gliogenic switch in neocortical radial glia cells is coordinated at the population level such that neurogenesis occurs from embryonic day 12 (E12) in mice until late embryonic development, and gliogenesis occurs from late embryonic development into early postnatal development. Once radial glia cells complete gliogenesis, they transform into astrocytes and lose their neural stem cell properties [4]. As a result, neurogenesis and gliogenesis occur as sequential and largely nonoverlapping processes during neocortical development [5]. The timing and mechanisms regulating the neurogenic-to-gliogenic switch in the neocortex is well-studied [5]. However, it remains unclear whether principles of neocortical development directly apply to non-neocortical regions of the cerebral cortex, such as the archicortex or hippocampus, of which development has been studied less extensively.

Thymidine analog methods were used in developmental biology research for decades to assay for proliferation and to determine the timing of cytogenesis across development [6,7,8,9,10,11]. Thymidine analogs incorporate into the DNA sequence during the S-phase of the cell cycle and can be used to assay proliferation at different time points after administration. Once incorporated into the DNA, the thymidine analog content gets diluted over subsequent cell divisions. It was estimated that 2–4 cell divisions dilute the thymidine analog content to undetectable levels [10,11]. However, if a cell is undergoing its final division before differentiating when it incorporates a thymidine analog, then it retains the thymidine analog label, which birth-dates the cell. Original birth-dating studies used a radioactive thymidine analog (^3^H-thymidine) to label dividing cells, which could be detected by autoradiography [11]. The second generation of thymidine analogs included 5′-bromo-2′-deoxyuridine (BrdU), 5′-iodo-2′-deoxyuridine (IdU) and 5′-chloro-2′deoxyuridine (CldU), which can be detected by antibody immunohistochemistry [7,8,9]. However, antibody detection of BrdU, IdU and CldU requires a caustic acid antigen retrieval step, which ruins the epitopes of many other proteins, making colocalization of cell type-specific markers challenging. The third generation of thymidine analogs, 5-ethynyl-2′-deoxyuridine (EdU), is widely used today and was modified to be compatible with Click chemistry, which utilizes a biochemical reaction to detect the EdU labeling and does not require a caustic acid antigen retrieval step [6].

The hippocampus can be subdivided into three regions, the Cornu Ammonis (CA) 1, the CA3 and the dentate gyrus (DG). Seminal studies by Bayer [12,13,14], Angevine [15] and colleagues used ^3^H-thymidine autoradiography to study the timing of neurogenesis in the developing hippocampus of rat and mouse, respectively. Collectively, these studies suggested that neurons in the CA1 and CA3 regions are generated embryonically until birth and that neurons in the DG have an extended generation time which continues postnatally. In fact, the DG maintains neural stem cells beyond development, which sustain neurogenesis throughout adulthood [16]. Multiple studies suggested that astrocyte generation in the hippocampus occurs during early postnatal development in rodents [17,18,19]. However, birth-dating techniques have never been used to directly determine the dynamics of astrocyte generation in the hippocampus and its subregions. Clonal lineage tracing studies showed that, during development, a single neural stem cell in the CA1 [20] or DG [21] germinal zone could generate both neurons and astrocytes for the corresponding region. However, the extended development of the DG raises the question of how neurogenesis and gliogenesis are coordinated within DG.

Here, we used thymidine-analog EdU birth-dating to investigate the timing and coordination of neurogenesis and astrogenesis in different subregions in the developing mouse hippocampus. Our results indicated that neurogenesis and astrogenesis in the CA1 and CA3 regions occur sequentially as nonoverlapping processes, similar to the neocortex, while astrogenesis in the DG completely overlaps with continuous neurogenesis in this subregion. In addition, we uncovered differential timelines of peak astrogenesis in each of the hippocampal subregions. Collectively, our results suggested that coordination of neurogenesis and gliogenesis in the DG does not mirror neocortical development and likely follows a distinct set of developmental principles.

## 2. Materials and Methods

### 2.1. Animals

All animal procedures used in this study were performed in accordance with protocols approved by the Institutional Animal Care and Use Committee of the University of Pennsylvania Perelman School of Medicine. The title of the protocol was “Molecular and cellular mechanisms regulating neural stem cells and neurogenesis”. The approval data was 2 May 2017. All mice used in this study were maintained on a C57BL/6 background. Animals were housed in a 14 h light/10 h dark cycle with food and water ad libitum. Both male and female mice were used for all experiments.

### 2.2. EdU Injection

EdU was administered as previously described [21]. A stock solution of 10 mg/mL EdU (Sigma, St. Louis, MO, USA, Cat. #900584) was prepared in normal saline solution (0.9%). EdU (50 mg/kg) was injected 2 times at a 6 h interval intraperitoneally into the pregnant female for embryonic time points (E12.5, E15.5, E17.5) or the pups for postnatal time points (P1, P3, P5, P7, P14), then brains were all analyzed at P30. For embryonic time points, the timed pregnancy was determined by identifying a vaginal plug (E0.5), and then EdU was administered intraperitoneally to the pregnant females on the target embryonic day. Samples from each time point came from different litters, because EdU injections into pregnant females affect the entire litter.

### 2.3. Tissue Processing, Click-iT EdU Labeling and Immunohistology

Animals were transcardially perfused with ice-cold Dulbecco’s phosphate-buffered saline (DPBS), followed by ice-cold 4% paraformaldehyde (PFA). Brains were fixed overnight in 4% PFA at 4 °C, and then cryoprotected in 30% sucrose solution overnight at 4 °C. Coronal brain sections (45 µm) were sliced using a sliding microtome (Leica, SM2010R). Every sixth section of each brain was collected into 1 of 6 wells in a 24-well plate containing anti-freeze solution (300 g sucrose, 300 mL ethylene glycol, 500 mL 0.1M PBS), and sections were stored at −20 °C.

Brain sections were stained as floating sections. Click-iT EdU labeling was performed according to manufacturer’s guidelines (Click-iT EdU Alexa Fluor 647 Imaging Kit, ThermoFisher Scientific, Cat. #C10340) prior to immunohistology. Brains were then washed in Tris-buffered saline (TBS) with 0.05% TritonX-100 and incubated in primary antibody solution (3.33% donkey serum and 0.05% TritonX-100 in TBS) overnight at 4 °C. Primary antibodies and dilutions used in this study included Rabbit anti-Prox1 (Abcam, Cat. #ab37128, 1:500 dilution), Mouse anti-neuronal nuclear protein (NeuN) Alexa Fluor 488 conjugated (Millipore, Cat. #MAB377X, 1:500 dilution), Rabbit anti-S100β (Agilent, Cat. #Z0311, 1:500 dilution) and Mouse anti-glial fibrillary acidic protein (GFAP) (Millipore, Cat. #MAB360, 1:1000 dilution). Brain sections were washed in tris-buffered saline (TBS) with 0.05% TritonX-100 and then incubated in secondary antibody solution (3.33% donkey serum and 0.05% TritonX-100 in TBS) and 4′,6-diamidino-2-phenylindole (DAPI) nuclear stain (Roche, 1:1000 dilution) for 1–2 h at room temperature. Alexa Fluor 488 and 555 secondary antibodies (Invitrogen, 1:250 dilution) were used. After a second set of washes, sections were mounted with 2.5% polyvinyl alcohol mounting medium with DABCO^®^ mounting media (Sigma, St. Louis, MO, USA, Cat. #10981).

### 2.4. Confocal Microscopy and Image Quantification

Brain sections across the anterior–posterior axis of the hippocampus were imaged as tiled z-stacks using a Zeiss LSM 810 confocal microscope (Carl Zeiss). Each tiled z-stack encompassed the entire hippocampus of a single brain section, and 3 anatomically matched brain sections were selected at equivalent levels across the dorso-ventral axis of the hippocampus and imaged for every sample. Either 20× or 40× objectives were used for imaging. Cells were considered positive for EdU when EdU label intensity was above background levels, regardless of the staining pattern within the nucleus. Images were analyzed and quantified using ImageJ software, and all cell counting was done blind to the timing of the EdU injection to ensure unbiased quantification. Quantification of the EdU^+^ cells generated at a particular developmental time point as a proportion of the total population of that cell type was performed in line with previous methodology from classic birth-dating studies [12,13,14]. Neurogenesis was quantified by counting the number of NeuN^+^EdU^+^ cells as a proportion of the total number of NeuN^+^ cells (CA1 and CA3), or by counting the number of NeuN^+^ prospero homeobox 1 (Prox1)^+^EdU^+^ cells as a proportion of the total number of NeuN^+^Prox1^+^ cells (DG). Astrogenesis was quantified by counting the number of GFAP^+^S100 calcium-binding protein B^+^(S100β^+^)EdU^+^ cells as a proportion of the total number of GFAP^+^S100β^+^ cells in a given region or subregion. For quantification, EdU cell labeling within animals and between animals was found to be very consistent throughout the quantitative analysis, as evidenced by consistent patterns of EdU labeling between regions within the same brain and low variability within groups.

## 3. Results

### 3.1. Developmental Neurogenesis in the Dentate Gyrus Is a Protracted Process Compared to CA Regions of the Mouse Hippocampus

**Figure 1:**
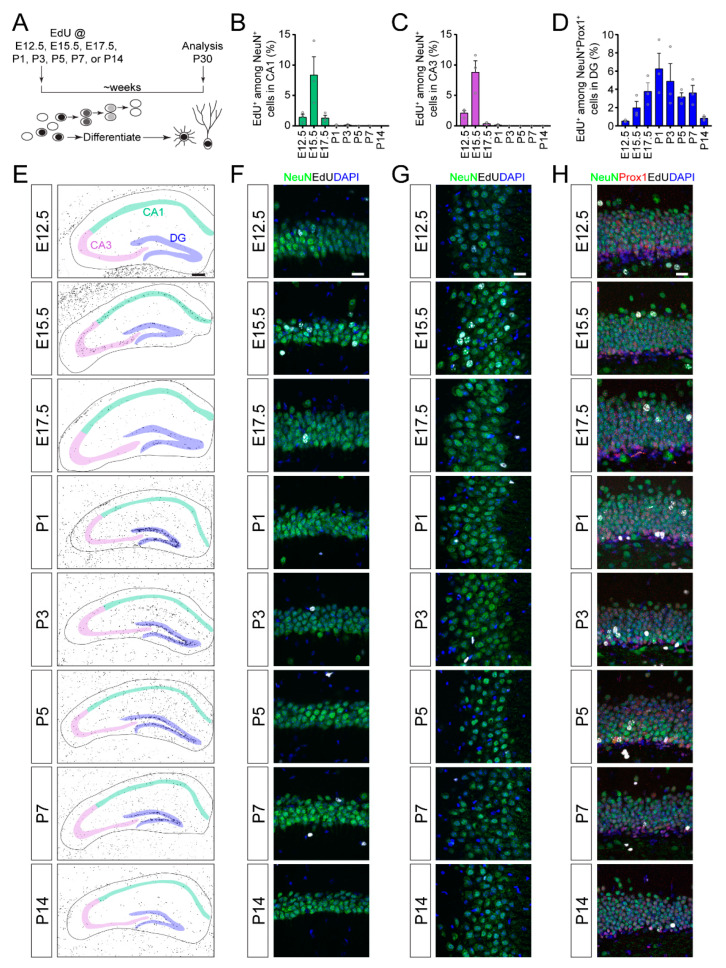
Neurogenesis in the developing mouse hippocampus. (**A**) A schematic illustration of the experimental paradigm for 5-ethynyl-2′-deoxyuridine (EdU) birth-dating of neurons and astrocytes. EdU was administered on a single day during development (E for embryonic day of gestation and P for postnatal day after birth) followed by a chase period until analysis at P30. (**B**–**D**) Quantification of neurogenesis in hippocampal subregions. Quantification of the proportions of NeuN^+^ pyramidal neurons in CA1 (**B**) or CA3 (**C**) regions, or the proportion of NeuN^+^Prox1^+^ dentate granule neurons in the DG (**D**) region that retained an EdU label at P30 from EdU injected at different times during development (*x*-axis). Values represent mean ± SEM, each sample value represented by a gray circle (*n* = 3 mice). (**E**) Black and white rendered images of EdU labeling (black) throughout the hippocampus at P30 when injected with EdU at the indicated time during development. Scale bar: 200 µm. CA1: cornu ammonis 1, CA3: cornu ammonis 3, DG: dentate gyrus. EdU^+^ neurons were quantified within the highlighted CA1, CA3 and DG regions for (**B**–**D**). (**F**–**H**) Sample projection confocal images of NeuN^+^EdU^+^ pyramidal neurons in CA1 (**F**) or CA3 (**G**) and NeuN^+^Prox1^+^EdU^+^ dentate granule neurons in DG (**H**) at P30 when injected with EdU at the indicated time during development. Scale bars: 20 µm. NeuN: neuronal nuclear protein.

We examined the timing of developmental cytogenesis in hippocampal subregions using an EdU pulse-chase, birth-dating technique [6,7,8,9] (Figure 1A). EdU was administered to mice on a single day of development at embryonic day 12.5 (E12.5), E15.5, E17.5, postnatal day 1 (P1), P3, P5, P7 or P14, and then chased to P30 when all analyses were conducted. EdU incorporates into cells in the S-phase of cell cycle at the time of administration, then, one of two things can happen: (1) Cells can continue to divide, diluting the EdU label to undetectable levels [10,11], or (2) cells can exit the cell cycle soon after incorporating the EdU, retaining the EdU label and indicating the birth-date of the cells. Thus, the EdU label-retaining cell population at P30 represents a snapshot of the cell types generated at the time of EdU administration (Figure 1E). We used colocalization of cell type-specific markers with EdU label-retaining cells to determine the timing of developmental neurogenesis and astrogenesis in the hippocampus.

First, we examined the timing of neurogenesis in the CA1, CA3 and DG regions of the hippocampus. We found that EdU-labeled pyramidal neurons (NeuN^+^EdU^+^) in the CA1 (Figure 1B,F) and CA3 (Figure 1C,G) regions were almost exclusively generated embryonically, with peak generation at E15.5. In contrast, EdU-labeled dentate granule neurons (NeuN^+^Prox1^+^EdU^+^) in the DG were gradually generated over embryonic and postnatal development, with peak neurogenesis occurring neonatally (Figure 1D,H). Thus, neurogenesis in the CA regions of the hippocampus occurs acutely during embryonic development, while neurogenesis in the DG is an extended and gradual process that peaks just after birth.

### 3.2. The Majority of Astrogenesis Occurs during the First Postnatal Week in All Regions of the Mouse Hippocampus

**Figure 2:**
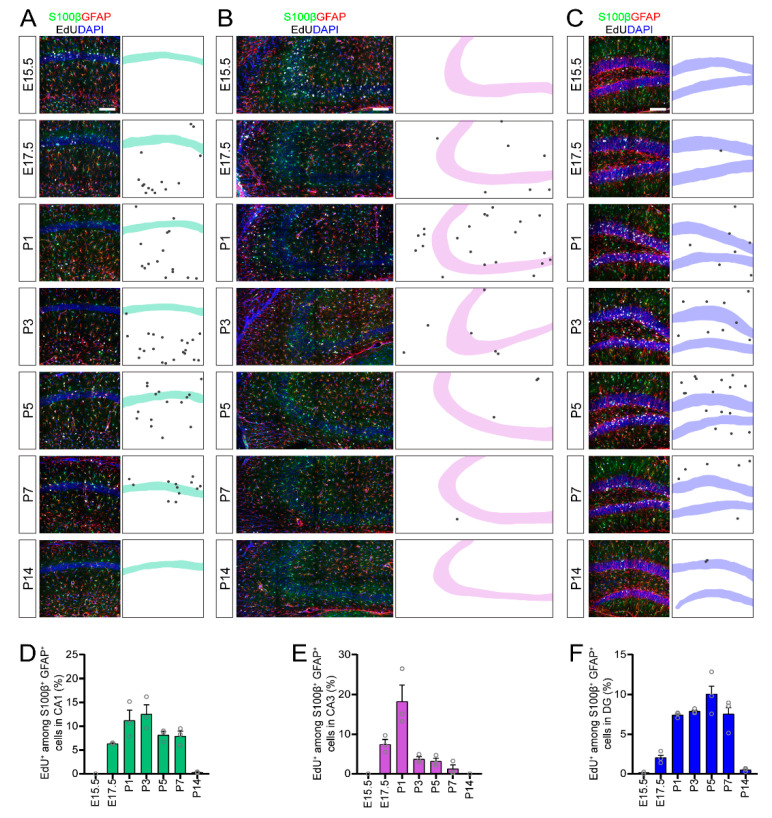
Astrogenesis in the developing mouse hippocampus. (**A**–**C**) Sample projection confocal images (left panels) and corresponding diagrams (right panel) of S100β^+^GFAP^+^EdU^+^ astrocytes in CA1 (**A**), CA3 (**B**) and DG (**C**) regions at P30 when injected with EdU at the indicated time during development. Black dots in the right panels highlight the location of S100β^+^GFAP^+^EdU^+^ astrocytes in the left panels. Scale bars: 100 µm. (**D**–**F**) Quantification of astrogenesis in hippocampal subregions. Quantifications of the proportion of S100β^+^GFAP^+^ astrocytes in whole CA1 (**D**), CA3 (**E**) and DG (**F**) regions that retained an EdU label at P30 from EdU injected at different times during development (*x*-axis). Values represent mean ± SEM, each sample value represented by a gray circle (*n* = 3 mice). GFAP: glial fibrillary acidic protein.

Next, we examined the timing of astrogenesis in the CA1, CA3 and DG regions of the hippocampus. We found that EdU-labeled astrocytes (S100β^+^GFAP^+^EdU^+^) were generated starting during late embryonic development (E17.5), but were predominantly generated during the first postnatal week in all regions of the hippocampus (Figure 2). Astrogenesis in the CA3 region occurred quite acutely, peaking around P1 (Figure 2B,E). Astrogenesis in the CA1 (Figure 2A,D) and DG (Figure 2C,F) regions occurred more gradually throughout the first postnatal week but differed in time-course. Astrocyte generation in CA1 peaked at the beginning of the first postnatal week, while astrocyte generation in DG peaked at the end of the first postnatal week (Figure 2D,F). Together, our data suggested that astrogenesis throughout the hippocampus collectively occurs from late embryonic development through the first postnatal week, but that that the time-course of peak astrogenesis depends on the hippocampal subregion.

### 3.3. Neurogenesis Overwhelms Astrogenesis at Every Stage of Dentate Gyrus Development

Neurogenesis gradually occurs over embryonic and postnatal development in the dentate gyrus, such that astrogenesis and neurogenesis occur simultaneously during neonatal development. Therefore, we wondered how the relative contribution of neuron and astrocyte generation changed over the course of development. We quantified the number of NeuN^+^EdU^+^ neurons in the granule cell layer (GCL) and the number of S100β^+^EdU^+^ astrocytes in the DG within the same brain sections and calculated the ratio of DG astrocytes: GCL neurons (S100β^+^EdU^+^ cells:NeuN^+^EdU^+^ cells) generated at each time point. We found that the number of neurons generated vastly outnumbered the number of astrocytes generated at every timepoint (ratio of DG astrocytes: GCL neurons: E15.5 = 0.01 ± 0.01, E17.5 = 0.08 ± 0.01, P1 = 0.12 ± 0.02, P3 = 0.20 ± 0.02, P5 = 0.38 ± 0.04, P7 = 0.29 ± 0.04, P14 = 0.09 ± 0.09; mean ± SEM, *n* = 3 animals per time point, value of 1 indicates equivalent numbers of astrocytes and neurons). There was a significant effect of developmental stage on the ratio of astrocyte to neuron generation (one-way ANOVA, F_(6,14)_ = 9.56, *p* = 0.0003), with a peak ratio at P5 of 0.38 astrocytes generated for every neuron generated. These results suggested that more neurons are generated than astrocytes in the DG across development, even during the period of peak astrogenesis.

### 3.4. Temporal Dynamics of Astrogenesis Vary in the CA1, CA3 and DG Subregions

**Figure 3:**
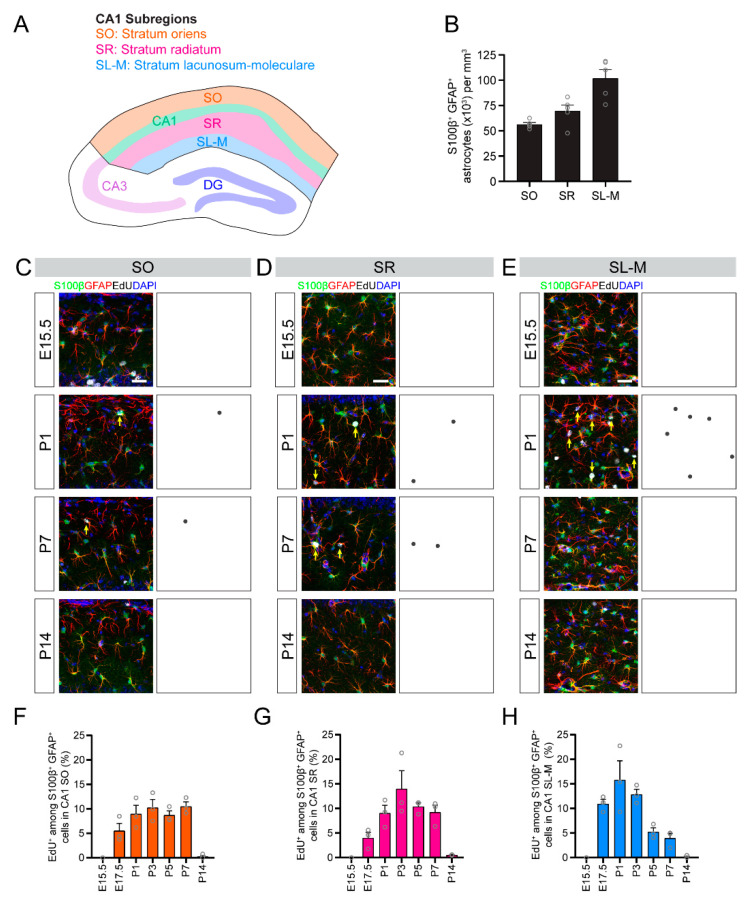
Astrogenesis in the developing CA1 subregions. (**A**) A schematic illustration of CA1 subregions. (**B**) Quantification of the S100β^+^GFAP^+^ astrocyte density in CA1 subregions. (**C**–**E**) Sample projection confocal images (left panels) and corresponding diagrams (right panel) of S100β^+^GFAP^+^EdU^+^ astrocytes in CA1 stratum oriens (SO; **C**), stratum radiatum (SR; **D**) and stratum lacunosum–moleculare (SL-M; **E**) at P30 when injected with EdU at the indicated time during development. Yellow arrows indicate S100β^+^GFAP^+^EdU^+^ astrocytes. Scale bars: 25 µm. (**F**–**H**) Quantification of astrogenesis in CA1 subregions. Quantification of the proportions of S100β^+^GFAP^+^ astrocytes in stratum oriens (**F**), stratum radiatum (**G**) and stratum lacunosum-moleculare (**H**) that retained an EdU label at P30 from EdU injected at different times during development (*x*-axis). Values represent mean ± SEM, each sample value represented by a gray circle (*n* = 5 mice in (B) and *n* = 3 mice in (**F**–**H**)).

Next, we examined the temporal dynamics of astrocyte generation in subregions of CA1, CA3 and DG. The CA1 region of the hippocampus is composed of the stratum oriens, pyramidal cell layer, stratum radiatum and stratum lacunosum-moleculare (Figure 3A). The majority of astrocytes are located in subregions outside of the CA1 pyramidal layer, and astrocyte density is dependent upon specific subregions (Figure 3B). We found that astrogenesis in different subregions of CA1 followed different time-courses. For example, astrocytes in the stratum oriens were consistently generated from E17.5 to P7 (Figure 3C,F), while astrocyte generation in the stratum radiatum was biased toward later timepoints (P3–P7; Figure 3D,G) and astrocyte generation in the stratum lacunosum-moleculare was biased toward earlier timepoints (E17.5–P3; Figure 3E,H).

**Figure 4:**
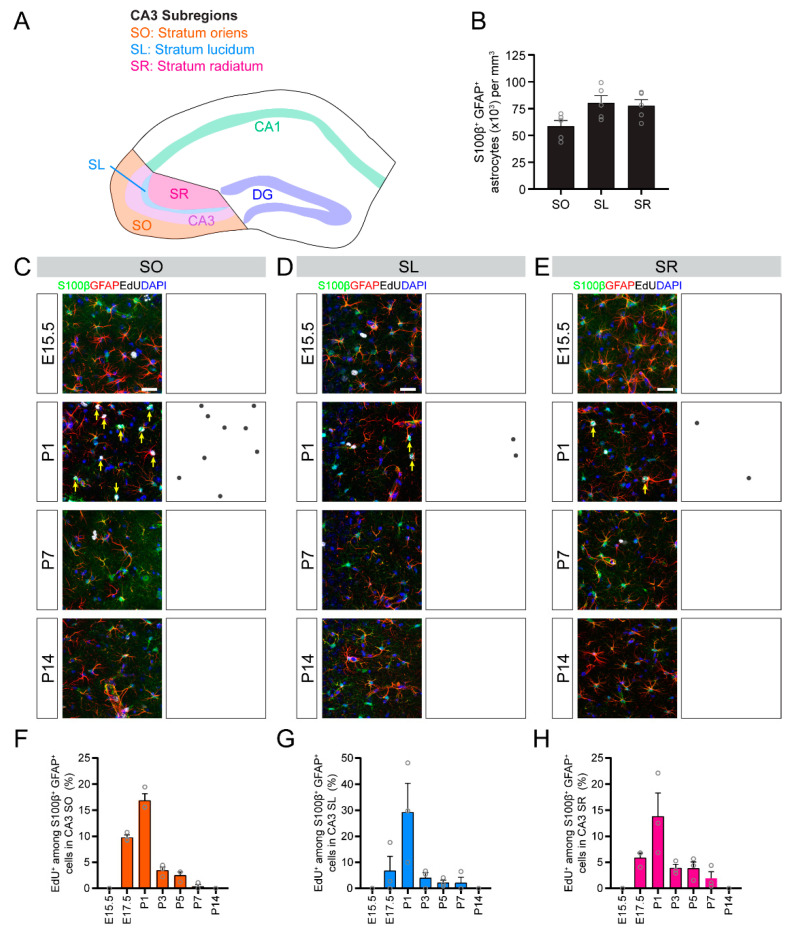
Astrogenesis in the developing CA3 subregions. (**A**) A schematic illustration of CA3 subregions. (**B**) Quantification of the S100β^+^GFAP^+^ astrocyte density in CA3 subregions. (**C**–**E**) Sample projection confocal images (left panels) and corresponding diagrams (right panel) of S100β^+^GFAP^+^EdU^+^ astrocytes in CA3 stratum oriens (SO; **C**), stratum lucidum (SL; **D**) and stratum radiatum (SR; **E**) at P30 when injected with EdU at the indicated time during development. Yellow arrows indicate S100β^+^GFAP^+^EdU^+^ astrocytes. Scale bars: 25 µm. (**F**–**H**) Quantification of astrogenesis in CA1 subregions. Shown are quantifications of the proportion of S100β^+^GFAP^+^ astrocytes in stratum oriens (**F**), stratum lucidum (**G**) and stratum radiatum (**H**) that retained an EdU label at P30 from EdU injected at different times during development (*x*-axis). Values represent mean ± SEM, each sample value represented by a gray circle (*n* = 5 mice in (**B**) and *n* = 3 mice in (**F**–**H**)).

The CA3 region of the hippocampus is composed of the stratum oriens, pyramidal cell layer, stratum lucidum and stratum radiatum (Figure 4A). As in the CA1 region, most astrocytes in the CA3 are located outside of the pyramidal cell layer and astrocyte density is dependent upon specific subregions (Figure 4B). We found that astrogenesis in all subregions of the CA3 followed a similar time course, with peak astrocyte generation occurring around P1 in the stratum oriens (Figure 4C,F), stratum lucidum (Figure 4D,G) and stratum radiatum (Figure 4E,H). Most astrocytes in the CA3 subregions were generated quite acutely compared with the more gradual generation of astrocytes in the CA1 and DG regions.

**Figure 5:**
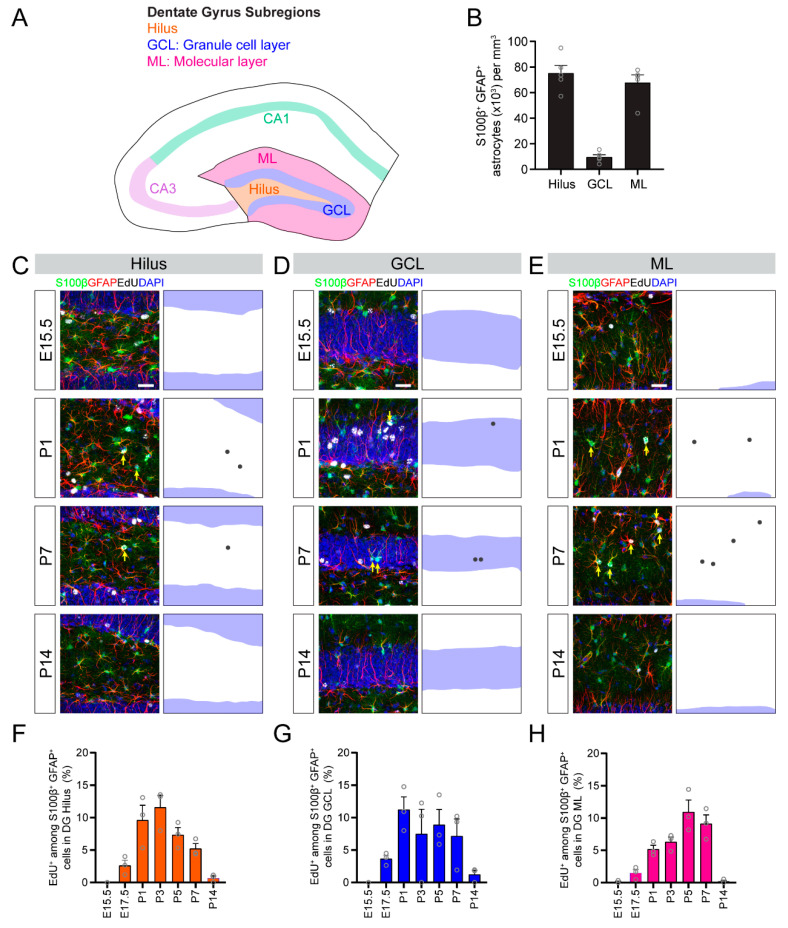
Astrogenesis in the developing dentate gyrus subregions. (**A**) A schematic illustration of dentate gyrus subregions. (**B**) Quantification of the S100β^+^GFAP^+^ astrocyte density in dentate gyrus subregions. (**C**–**E**) Sample projection confocal images confocal images (left panels) and corresponding diagrams (right panel) of S100β^+^GFAP^+^EdU^+^ astrocytes in the hilus (**C**), the granule cell layer (GCL; **D**) and the molecular layer (ML; **E**) at P30 when injected with EdU at the indicated time during development. Yellow arrows indicate S100β^+^GFAP^+^EdU^+^ astrocytes. Scale bars: 25 µm. Blue shade indicates granule cell layer (**F**–**H**) Quantification of astrogenesis in dentate gyrus subregions. Quantifications of the proportion of S100β^+^GFAP^+^ astrocytes in the hilus (**F**), the dentate granule cell layer (**G**) and the molecular layer (**H**) that retained an EdU label at P30 from EdU injected at different times during development (*x*-axis). Values represent mean ± SEM, each sample value represented by a gray circle (*n* = 5 mice in (**B**) and *n* = 3 mice in (**F**–**H**)).

Finally, we examined the temporal dynamics of astrogenesis in the DG region of the hippocampus. The DG can be divided into three subregions, namely, the hilus, the granule cell layer and molecular layer (Figure 5A). Most astrocytes in the DG are located in the hilus and molecular layer regions (Figure 5B). We found that the timing of astrocyte generation in the DG starkly varied by specific subregions. Astrocytes in the hilus were predominantly generated between P1–P3 (Figure 5C,F), while most astrocytes in the molecular layer were generated later between P5–P7 (Figure 5E,H). The small population of astrocytes in the granule cell layer, not including the radial glia-like neural stem cells, were gradually generated over the course of the first postnatal week (Figure 5D,G).

In summary, the timing of neurogenesis and astrogenesis in CA1 and CA3 regions of the hippocampus follow the sequential, nonoverlapping properties of the neocortex, while the extended nature of neurogenesis in the DG obscures a clear neurogenic-to-gliogenic switch at the population level.

## 4. Discussion

**Figure 6:**
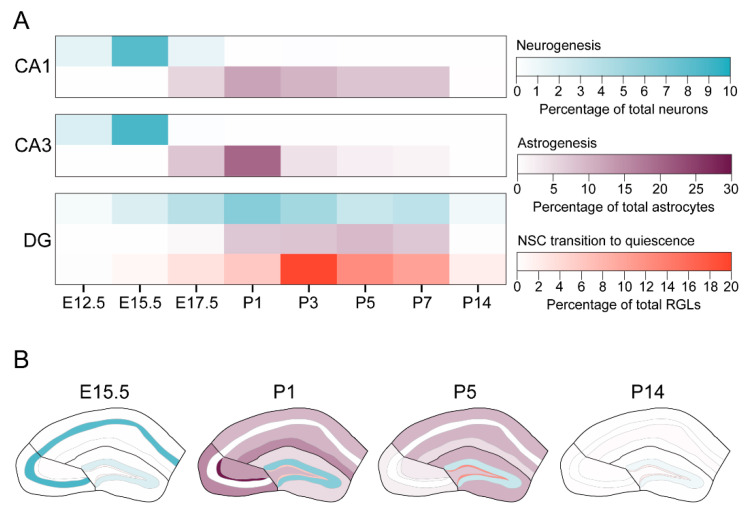
The timing of developmental neurogenesis and astrogenesis by hippocampal subregion. (**A**) Heatmap diagrams summarizing the developmental timing of neurogenesis (blue) and astrogenesis (purple) in the CA1, CA3 and dentate gyrus of the mouse hippocampus. Neurogenesis and astrogenesis occur sequentially and are largely nonoverlapping in the CA1 and CA3 regions. In contrast, neurogenesis and astrogenesis occur simultaneously in the dentate gyrus, and at the same time that dentate gyrus neural stem cells (NSCs) transition into quiescence (red). NSC transition to quiescence data are from Berg et al., 2019. (**B**) Heatmap diagrams summarizing the developmental timing of neurogenesis (blue) and astrogenesis (purple) in subregions of the CA1, CA3 and dentate gyrus, as well as the NSC transition to quiescence in the subgranular zone of the dentate gyrus (red). Colors correspond to the same legend in (**A**). Neurogenesis in the CA1 and CA3 regions dominates during embryonic development. Neurogenesis in the dentate gyrus and astrogenesis throughout the hippocampal regions occurs during early postnatal development, but the timing of peak astrogenesis varies by subregion. In addition, dentate gyrus NSCs in the subgranular zone transition into quiescence during early postnatal development. Finally, most cytogenesis in the hippocampus has ceased by P14, except for low levels of neurogenesis and astrogenesis in the dentate gyrus, which continues throughout adulthood.

Our study used an EdU pulse-chase birth-dating technique to reveal the temporal dynamics of neurogenesis and astrogenesis in the developing mouse hippocampus and uncovered regional differences in the timing and coordination of neuron and astrocyte generation during embryonic and early postnatal hippocampal development. While many fundamental developmental processes were studied extensively in the neocortex, little is known about how neurogenesis and astrogenesis occur in the developing mammalian hippocampus. Our findings provide a comprehensive resource outlining the timing of developmental neurogenesis and astrogenesis in hippocampal subregions. In addition, by comparing the timing of neurogenesis and astrogenesis in the same experiment, we could directly study the coordination of these developmental processes, unlike many previous studies which focused individually on neurogenesis or astrogenesis [12,13,14,15,17,18,19]. Our findings reveal that the timing of peak astrogenesis during early postnatal development differs by specific hippocampal subregions (Figure 6). In addition, neurogenesis and astrogenesis in the CA1 and CA3 regions occur sequentially, similar to the neocortex. In contrast, astrogenesis completely overlaps with the extended neurogenesis that occurs in the DG, such that developmental neurogenesis and astrogenesis occur simultaneously. Together, our results uncover new insights into hippocampal astrocyte generation and suggest a unique coordination of neurogenesis and astrogenesis in the DG region of the hippocampus.

By mapping a time course of cytogenesis that spans both embryonic and postnatal development, our study could not use littermate comparisons between time points. EdU cannot practically be administered to individual embryos in utero, but instead must be administered to the entire litter by EdU administration to the dam. As a result, we could not administer EdU to littermates at different developmental timepoints. This technical constraint could potentially introduce some limitations to our study. First, it is possible that litter-to-litter variation could contribute to observed differences in cytogenesis. Second, it is possible that differences in the pharmacokinetics of EdU in embryos compared to postnatal pups could also contribute to the observed differences in cytogenesis. We used multiple high doses of EdU to achieve a saturating dose, which would be less vulnerable to subtle differences in pup-rearing between litters, and EdU pharmacokinetics between the in utero versus postnatal ages. Because we were interested in the temporal dynamics of neurogenesis and astrogenesis, rather than in quantitative measures of cell genesis, we believe that the limitations of out experimental paradigm did not greatly impact the conclusions of our study.

Though previous studies approximated the timing of astrogenesis in the hippocampus based on the appearance of stellate astrocytes during development [17,18,19], our study used EdU incorporation and molecular markers to directly birth-date astrocytes in the hippocampus. As a result, we provided a comprehensive timeline of astrogenesis in each hippocampal subregion and revealed fundamental properties of hippocampal astrocyte generation. First, our findings suggested that astrocytes are generated in a region-specific order, such that astrogenesis first begins in the CA3 region, followed by the CA1 and DG regions (Figure 6A). In addition, subregions within the CA1 and DG regions follow distinct temporal patterns of astrogenesis (Figure 6B). For example, astrocytes in the stratum lacunosum-moleculare are generated earlier than astrocytes in the stratum radiatum of the CA1 region, and astrocytes in the hilus are generated earlier than astrocytes in the molecular layer of the DG region. Little is known regarding how astrocytes are generated during hippocampal development and, as a result, fundamental principles of astrogenesis in the hippocampus remain unknown. Previous work showed that spatially segregated germinal zones in the medial pallium give rise to neurons in the CA1, CA3 or DG hippocampal subregions, suggesting that NSCs in these distinct germinal zones are regionally specified [21,22]. However, it is unknown whether a single precursor can generate astrocytes for multiple hippocampal subregions or if astrocyte precursors are restricted to a single hippocampal subregion. In addition, though it is assumed that neural precursors that give rise to neurons in the CA1, CA3 or DG subregions also give rise to astrocytes in those regions, the source of hippocampal astrocytes was not explicitly identified. Our data suggested that there are regional differences in the timing of astrogenesis in the hippocampus, which could be a result of the source of astrocyte precursors, the timing of the neurogenic-to-astrogenic switch or the astrocytic requirements of different hippocampal subregions. Future studies should explore the basic principles of astrocyte generation in the hippocampus and how astrocyte generation is coordinated between hippocampal subregions.

By comparing neuron and astrocyte generation using the same methodology in a single experiment, our study captured the coordination of neurogenesis and astrogenesis within each hippocampal subregion. Much of what we know about the coordination of neurogenesis and astrogenesis comes from studies of neocortical development, in which neurogenesis occurs embryonically, followed sequentially by astrogenesis during early postnatal development [3,4]. Our data demonstrate that these fundamental properties of neocortical development apply to the CA1 and CA3 regions of the hippocampus, where we see a similar coordination and timing of neurogenesis and astrogenesis. Strikingly, development of the DG region of the hippocampus does not follow many principles of neocortical development. First, we found that neurogenesis in the DG is a gradual and protracted process. Though the onset of neurogenesis in the DG occurs during early embryonic development, similar to the neocortex and CA regions, DG neurogenesis occurs more gradually over a longer period of time that extends into postnatal life. In contrast, neurogenesis in the neocortex and CA regions occurs more rapidly and is completed by birth. Second, we found that neurogenesis and astrogenesis occur simultaneously during DG development. The majority of astrogenesis in the DG occurs during the first postnatal week, similar to that of the neocortex and CA regions of the hippocampus. However, unlike the neocortex and CA regions, in which neurogenesis and astrogenesis are temporally segregated, neurogenesis in the DG continues through and beyond the postnatal period of astrogenesis (Figure 6A). As a result, there does not appear to be a population-wide, neurogenic-to-gliogenic switch in the DG. Throughout the stages, neurogenesis overwhelms astrogenesis, even during the peak of astrogenesis in the DG. Interestingly, active neurogenesis and astrogenesis within DG occurs concurrently with the transition of neural stem cells into quiescence [21] (Figure 6A). Together, these findings prompt a multitude of questions. Does a subpopulation of neural stem cells undergo a neurogenic-to-gliogenic switch to generate astrocytes, while another subpopulation continues engaging in neurogenesis postnatally? Does the neurogenic-to-gliogenic switch occur at the single cell level or do neural precursors in the DG remain multipotent during the overlap of neurogenesis and astrogenesis? What mechanisms initiate and terminate developmental astrocyte generation in the DG? The answer to some of these questions may be intertwined with the fact that the DG maintains neural stem cells beyond development, which sustain neurogenesis throughout adulthood (Figure 6) [16,21]. Future research to uncover the basic properties of DG development, which seem to defy conventional principles of developmental neurobiology, would broaden our understanding of mammalian brain development.

## 5. Conclusions

We provide a comprehensive timeline of neurogenesis and astrogenesis in mouse hippocampal subregions that could serve as a foundation for future studies studying hippocampal development. We also uncovered fundamental insights into regional differences in the timing and coordination of neurogenesis and astrogenesis within the hippocampus and identified unique features of DG development that challenge widely accepted principles of developmental neurobiology.
